# Psychopathology, trauma and delinquency: subtypes of aggression and their relevance for understanding young offenders

**DOI:** 10.1186/1753-2000-5-21

**Published:** 2011-06-29

**Authors:** Hans Steiner, Melissa Silverman, Niranjan S Karnik, Julia Huemer, Belinda Plattner, Christina E Clark, James R Blair, Rudy Haapanen

**Affiliations:** 1 Stanford University School of Medicine, Department of Psychiatry and Behavioral Sciences, 401 Quarry Road, Stanford, California, 94305, USA; 2 University of Chicago, Department of Psychiatry & Behavioral Neuroscience, Chicago, Illinois, USA; 3 Medical University of Vienna, Department of Child and Adolescent Psychiatry, Vienna, Austria; 4 Kinder- und Jugendpsychiatrischer Dienst des Kantons Zürich, Zürich, Switzerland; 5 University of Washington, Seattle, Washington, USA; 6 National Institute of Mental Health, Washington, District of Columbia, USA; 7 University of California, Davis, California, USA

## Abstract

**Objective:**

To examine the implications of an ontology of aggressive behavior which divides aggression into reactive, affective, defensive, impulsive (RADI) or "emotionally hot"; and planned, instrumental, predatory (PIP) or "emotionally cold." Recent epidemiological, criminological, clinical and neuroscience studies converge to support a connection between emotional and trauma related psychopathology and disturbances in the emotions, self-regulation and aggressive behavior which has important implications for diagnosis and treatment, especially for delinquent populations.

**Method:**

Selective review of preclinical and clinical studies in normal, clinical and delinquent populations.

**Results:**

In delinquent populations we observe an increase in psychopathology, and especially trauma related psychopathology which impacts emotions and self-regulation in a manner that hotly emotionally charged acts of aggression become more likely. The identification of these disturbances can be supported by findings in cognitive neuroscience. These hot aggressive acts can be delineated from planned or emotionally cold aggression.

**Conclusion:**

Our findings support a typology of diagnostic labels for disruptive behaviors, such as conduct disorder and oppositional defiant disorder, as it appears that these acts of hot emotional aggression are a legitimate target for psychopharmacological and other trauma specific interventions. The identification of this subtype of disruptive behavior disorders leads to more specific clinical interventions which in turn promise to improve hitherto unimpressive treatment outcomes of delinquents and patients with disruptive behavior.

## Introduction

One of the potentially most fruitful contributions of developmental psychiatry to human health is the study of delinquent populations. In the past decade, it has become clear from studies in different countries and continents [1-10] that delinquents, (ie. adjudicated youth), are a highly psychiatrically morbid population in dire need of services. This is especially true for psychiatric trauma related psychopathologies among young offenders with clear evidence of high rates of Posttraumatic Stress Disorder and Dissociative Disorder [11-13].

Such psychopathology is not insignificant or inconsequential, as it seems to persist months into incarcerative experiences. These psychopathologies also put these young people at risk for the most dire immediate outcomes, in addition to maladaptive developmental trajectories and increased criminal recidivism [14]. Finally, we suspect that the persistence of such psychopathologies contribute significantly to what has been described as the "cycle of violence" in the criminological/epidemiological literature [15]. Psychiatrists as well as other mental health professionals are probably in an excellent position to contribute to disrupting the perpetuation of acts of aggression from generation to generation by providing effective treatment of these pathologies.

In this paper, we argue that there are sufficient findings from a series of international studies supporting a trauma related psychopathology specific pathway into and, hopefully, out of juvenile crime. These findings also have implications for the taxonomy of disruptive behaviors and most likely will alter hitherto modest successes in the rehabilitation of juvenile offenders. We have consistently put forward this argument in previous presentations and publications, especially due to our experience as consultants to the California Youth Authority [16].

In the study of juvenile delinquency, we are immediately brought face to face with a paradox: on one hand, problems with disruptive behavior are extremely common in child psychiatric clinics [17-19]. On the other hand, in comparison to problems with attention regulation and even pediatric anxiety and depression, our database is much more restricted when considering young offenders. In an important first step to ameliorate this situation, the DSM and ICD systems introduced diagnostic labels addressing problems of aggression and disruptive behavior from the vantage point of clinical medicine as early as 1980 (DSM-III) [20]. This action corrected a deficiency in the mental health sciences, which up until then, and even somewhat since, has shown a curious disregard for disorders of anger, hostility, aggression and other antisocial behavior. This omission likely reflects the psychiatric pioneers' greater interest in disorders of anxiety, mood, and problems with reality-testing. The introduction of diagnostic labels like conduct disorder and oppositional defiant disorder achieved, for the first time, an important step in the scientific/medical approach to problems of delinquency because they separated diagnosis and treatment from adjudication. This new labeling permitted early identification, preventive intervention and treatment outside the algorithms and confines of the juvenile justice system; a desirable outcome, as these systems are fraught with their of problems and inconsistencies. These labels also re-focused the basic neurosciences on more concerted efforts to delineate the underpinnings of these disorders of aggression [21].

The history of the study of aggression from a psychiatric/scientific perspective is therefore a relatively modern one, beginning in the 20^th ^century with the work of August Aichhorn (1925) in Vienna [22]. Aichhorn sought to bring the intra-psychic world described by Freud and others, as an explanatory tool to the distinctly social/criminal acts that he witnessed among delinquents. His study published under the title "Wayward Youth" forms one of the key scholarly pieces in the study of modern aggression and marked the beginning of a synthetic approach by bringing in the clinical point of view; delinquents could be viewed as patients, sufferers. Those who inflicted harm on others could be approached from a medical/psychological perspective. His book contains case histories especially in the third chapter, which when stripped of their local Viennese color, stand as examples of delinquent youths in the modern Industrialized Western nations, as they struggle with highly traumatic events, such as parental death, threats to their own lives and abusive parenting.

Other landmark studies brought in the impact of social isolation and displacement in the genesis of antisocial behavior. John Bowlby [23] utilized the British relocation of youths into the countryside during World War II to study the plight of young people and their propensity to become criminals in the wake of dislocation from home and while struggling with separation from their families of origin. "Forty-Four Juvenile Thieves: Their Character and Home Life" in 1944 links traumatic events surrounding separation to the development of antisocial and aggressive behavior [24]. This line of research connected the emergence of disruptive behavior to the occurrence of life changing events.

Expanding on these ideas, two other pioneers, Fritz Redl and David Wineman put forward a set of ideas in "Children Who Hate: The Disorganization and Breakdown of Behavior Controls" (1951) about re-socialization of aggressive youths [25]. Redl did not believe that counseling or psychotherapy were sufficient to effect change for youth, and instead sought to create a new therapeutic milieu within which children could learn about their behaviors and then change them. This thinking was in line with Aichhorn, who thought that aggression was a normative phenomenon that yielded to positive developmental influences. Such thinking also connects with the insights from ethology [26] that found that aggression has an adaptive purpose and can be shaped developmentally in a pro-social context, and further redirected and refined. The basic assumptions of this philosophy continue to be found in modern residential programs [27] and certainly inform the theories of criminological treatment and rehabilitation [28]. As we shall see below, the planned, instrumental, proactive (PIP) subtype of aggression is a good candidate for such treatment, as there are currently (few if any) other interventions that can affect such complex behaviors. Medications, short of rendering the patient unconscious, are only modestly effective against such complex behaviors which run on multiply layered neuroarchitectures. From these early beginnings, there is a thread of studies up until the present that repeatedly document the impact of environmental adversity in many different forms as being highly relevant to the genesis of maladaptive aggression [16].

At the same time, other authors have pursued the idea that there are a set of intrapersonal factors which puts the individual at risk for problems with maladaptive aggressive behavior. Ever since the classic monograph by Hervey Cleckley [29], studies of genes, heart rate, galvanic skin response, cortisol and many other indicators of arousal under duress have documented the fact that in certain individuals, with maladaptive patterns of aggression, stress reactivity is reduced across all channels of expression [30-34]. The term "Psychopathy" popularized by Cleckley, seeks to delineate those that struggle with repeatedly committing such calculated acts, while demonstrating little remorse. Recent imaging studies are beginning to identify the CNS pathways in adolescent individuals with callous-unemotional traits which appear quite distinct from areas of the brain affected with more impulsive, reactive aggression [21]. Thus, while the observable outcome may be similar in terms of descriptive behavior, the neurobiological underpinnings of people committing aggressive acts in the context of psychopathy are distinct from those who react aggressively to a perceived of imagined threat. Recent epidemiological studies of youths in a 2-year prospective design also point in the same direction [35,36], prompting the authors to call for inclusion of a diagnostic subdivision on the basis of callous-unemotional traits.

We would like to further support these efforts by summarizing data from another dimension; emotionally charged aggression which seems to have a special relationship to psychiatric disorders of trauma [37].

## A Very Old and New Division for Disorders of Maladaptive Aggression

In the law, there has been a long standing distinction between crimes of passion or crimes of malice and forethought. This distinction is present in all cultures and has endured over thousands of years. The bifurcation in pertinent neuro-scientific findings lends new support to this distinction. At the present time, our existing taxonomy does not reflect these distinctions which capture the processes by which aggressive acts come to be [17,18]. Oppositional Defiant Disorder, Conduct Disorder and Intermittent Explosive Disorder, the paraphilias and sexual disorders involving aggressive acts do not specify whether these symptoms are generated in emotionally-charged or carefully planned psychological states [38].

This lack of distinction leads to a within-class heterogeneity that in turn renders these diagnostic labels less useful. Disruptive behavior disorders are co-morbid with disorders as wide ranging as substance dependence, mental retardation, autism, PTSD, bipolar disorder and depression [39]. This heterogeneity of diagnostic categories is increasingly problematic in an era of developmental psychiatry where we are acquiring increasingly specific treatment methods for specific disorders. After having diagnosed someone with conduct disorder, the clinician is still left with questions as to which treatment would be most appropriate. This is partly a function of the relatively limited number of clinical trials in this population [40] but also a result of having two very different sets of symptoms under one set of diagnostic caregories.

Could we improve our approach to disruptive behaviors by seriously considering an emotional/trauma specific form of aggression that is distinct from disorders generated predominantly by deficient arousal, empathy and self-regulation? Using recent progress in the cognitive neurosciences, we propose a new theoretical framework for psychiatric approaches to aggression and anti-sociality and report some results that test this new framework in populations with high ecological validity.

Over the last few decades there have been attempts to subdivide aggressive behavior, which have been well described in criminological and more recently in the developmental psychiatry literature [41]. Table 1 shows a summary of the many ontological categorical divisions of antisocial/disruptive behavior that have been made by various investigators and researchers of aggression [42-49].

**Table 1:** Empirically Supported Subtypes of Aggression

Subtypes of Aggression	Predominant Empirical Support
Overt/Oppositional/Covert [42]	Prospective, developmental, human
Reactive/Proactive [43]	Prospective, developmental, human
Affective/Predatory [44]	Experimental, clinical, developmental, human
Defensive/Offensive [45]	Experimental, animal
Socialized and Under - Socialized [46]	Clinical, developmental, human
Impulsive/Controlled [47]	Forensic, clinical, human
Hostile/Instrumental [48] Impulsive/Premeditated [49)	Clinical, experimental, developmental Forensic, adult, clinical, experimental

Across investigators, these categories generally share a two-part division which can be broadly grouped; acts of reactive, affective, defensive and impulsive aggression, on one hand, and acts of proactive, instrumental and planned aggression [50]. By relabeling the first grouping as emotionally "hot" aggression, we can combine the descriptors of this label into a new acronym (RADI). These are acts of unplanned, very often overt aggression. The perpetrator anticipates a potentially negative outcome of a situation, but feels the need to act aggressively to avert a negative outcome (such as being attacked), while understanding that his acts are outside of the social norm. The triggering and perpetuating emotions are almost uniformly negative and run the gamut from fear, disgust, contempt, to sadness, rage, and frustration. Following the event, the perpetrator knows that he or she has done wrong and is usually contrite, assuming responsibility for the actions without necessarily knowing why he or she acted in the manner that they did.

On the other side of the taxonomy are acts of aggression that are but one form of instrumental behavior [21]. These acts are carefully planned, very often covert and they are viewed in a positive light be the perpetrator, who anticipates a positive outcome (such as acquisition of goods or territory, or improved social standing). The triggering/perpetuating emotions are usually muted, but can be positive: interest, even happiness. The labels generate the acronym PIP, designating emotionally "cold" aggression [50]. Implicit in this model is the fact that all these forms are part of a normal human repertoire of behavior that facilitates survival [26]. There is nothing intrinsically pathological about either form of aggression provided they occur in an appropriate context. RADI aggression is useful in defending one's own under threat; PIP aggression leads to positive outcomes in highly competitive situations. PIP aggression may be adaptive on Wall Street and in other extremely competitive settings. It is only when RADI and PIP occur in a clustered forms, are out of context, are unusually severe and disproportionate to their trigger, or do not cease once the other has signaled defeat that they alert a clinician's attention to look for more signs of psychopathology [41].

## The Neuroscience of Hot and Cold Aggression

Recent research in the neuroscience of aggression support the division into PIP and RADI subtypes [21]. Findings regarding the two forms in imaging and cognitive neuroscience provocation studies point to the fact that these two forms of aggression run on different neuro-architectures. These have been discussed these in great detail in other publications [21,51,52] and will only be briefly summarized.

In both forms of aggression, we see structures that serve as activators and regulators for the aggressive acts. In both forms, it is likely that these architectures stand in a homeostatic balance. Pathology can result if there is excessive activation, deficient down-regulation or both. Defects in the system most likely can be induced by endogenous (e.g. constitutional, genetic) factors, or exogenous factors, such as trauma, deficient nutrition, brain damage, etc,; or a combination of both. Concerning the architectures related to hot RADI aggression, work with humans and animals have identified a distinct, hardwired circuit, present from very early development upon which forms the basis for the activating arm of hot aggression. The circuit is part of the threat response system and runs from the medial nucleus of the amygdala to the medial hypothalamus and from there to the dorsal half of periaquaeductal gray. Controlling and down-regulating structures that have been identified are in the anterior cingulated, the ventrolateral and orbital-prefrontal cortex. The system reacts to threat and fear inducing stimuli in a modular fashion: low doses of threat result in freezing. Increasing levels of threat results in flight. The final response is fight - rearing up, when the animal finds itself trapped in conditions of inescapable threat. This last and final step is perhaps closest to the situation that humans find themselves in during severely abusive or life-threatening situations, and where escape is impossible (e.g. immaturity and dependency). These structures can become dysregulated [21] by facilitating emotional activation, to the point where they overwhelm the capacity of the regulating structures to contain emotional activation. (A predominantly exogenous case in point would be traumatic emotional discharge; an endogenous example the excessive activation present on a genetic basis in a bipolar patient). Dysregulation can also occur when there are endogenous or exogenous impediments in the controlling structures (as might be the case in traumatic brain injury along the lines of the classical case of Phineas Gage; or in certain forms of autism). Damage to the basic threat circuits in the relevant frontal lobe regions has been shown to increase the risk of RADI aggression in children [53] and adults [54]. In a recent study of conduct disordered youth with an extensive history of trauma, our research group found that these youth often conflated the experiences of sadness, fear and anger [55]. This lack of ability to differentiate these emotional states goes to the heart of the functionality threat response system and may explain why these youth express higher levels of RADI aggression when functioning under moderate levels of duress. Emotions are not distinct experiences, and they do not lead to emotion specific action. Any stress can be perceived as threat if the relevant control circuit is damaged and activates the self-defense system.

In contrast, the neuro-architectures supporting PIP or cold aggressive acts seem to run on a wider network of less hard-wired circuitry, perhaps not fully present early in development, but slowly formed under the influence of shaping social forces. Utilizing multiple structures that stand in more flexible interplay [21,51,52] cold aggressive behaviors are similar to other forms of instrumental behaviors, such as deceit, which appears to draw widely on diverse brain resources to accomplish a very complex task. The planning of the aggressive act, the consideration of the proper timing and context, the consideration for disguise and escape all involve careful action which is usually not done well in a state of high negative emotional charge. It is difficult to be impulsive while carrying out the heist of diamonds from the Topkapi Museum, to conjure up a grand cinematic example. The most appropriate animal model for PIP is the cat laying in wait for the mouse to appear out of her domicile. The cat is focused, calm, ready to jump, not frightened, angry and sad. Most recently, there have been fMRI studies suggesting that in adolescents with callous unemotional traits, the connection between the emotional amygdala respond less to others fearful faces, but not in angry and normal faces [52]. In a similar finding, Popma et al [32] showed that some children with disruptive behavior disorders showed decreased reactivity in a range of emotional activation channels (self report, cortisol, heart rate). Karnik et al. [33] reported, that in incarcerated older males, heart rate and self reported response to a standardized speech task was significantly lower than in age matched normal adolescents. Interestingly, it was also found that younger boys in juvenile hall who were still living under conditions of continuous threat showed elevated heart rates, as one would expect from children who are being actively traumatized. These findings remained significant after controlling for age effects [33].

On the side of regulatory structures, a recent finding in an fMRI study of 42 children with psychopathic traits (mean age 14, range 10 to 17) reports [51] that these children have abnormal ventro-medial prefrontal cortex responsiveness during a Reversal Learning Task. These effects were maintained while controlling for the presence or absence of ADHD. In contrast to normal and ADHD adolescents, these individuals with psychopathic traits persisted in a losing strategy during their reversal learning task, instead of shifting sets as the other children did. This deficit if confirmed in a larger scale study could relate to the "inability to learn from experience" that is often observed in psychopathic individuals.

## Scaling up The Model: Looking at Larger Samples

While the neuroscience studies of aggression have yielded exciting and potentially useful findings of hot and cold aggression, the challenge remains that most of these studies have limited sample sizes mostly due to the present research techniques involving functional neuro-imaging. Laboratory based studies always leave open the question of external validity, especially when working with delinquent populations. The question that arises is whether PIP and RADI forms of aggression can be used to effectively separate clinical and non-clinical samples. Do these two forms of aggression present differently; are there correlates of clinical significance? What is the degree of their overlap, and how much does one form predict the presence of the other? Finally, can we employ this distinction to clinical trials and show that they make a difference?

## Measuring Radi and Pip Aggression

To enable researchers and clinicians to use the proposed sub-typing of aggression, tools are needed to accurately and consistently assess the presence of PIP and RADI aggression. At the present time, there is no single diagnostic tool that spans the entire age range and measures both of these constructs. Instruments do exist that capture either one or the other of these typologies [18,41], but not all have been used extensively, across the life span, and most of them have found limited use in incarcerated youth populations.

A potential solution to this methodological problem is the utilization of well-established screening instruments for youths that contain related constructs. Evidence is developing that suggests that the existing and widely used diagnostic system developed by Achenbach and colleagues contains the two subtypes under different labels. The Child Behavior Checklist (CBCL) and its companion tool the Youth Self-Report (YSR) [56] both assess dimensions of "aggressive behavior" and "delinquent behavior" within its subscales in the version of 1999-2000. These scales were later relabeled in 2001. Ligthart and colleagues (2005) have reported that the CBCL (for 4-18 year olds) seems to contain two factors which they identified as "relational" and "direct" aggression [57]. In their study of over 7000 7-year old twin pairs using a principal components analysis, they were able to identify these subtypes. In boys they found a correlation between the two subtypes of 0.56 and 0.47 for girls. Boys appeared to score higher for both types of aggression. These findings fit within our emerging understanding of PIP and RADI aggression. In this schema, relational aggression would fall under PIP while direct aggression corresponds to RADI.

This finding is supported by other previous research. In a study by Achenbach et al. [58], experts rated CBCL items for consistency with the diagnostic categories of the Diagnostic and Statistical Manual of Mental Disorders [59], thus combining empirical and diagnostic approaches. Five out of six items of the direct aggression factor were found to describe symptoms of conduct problems, while none of the items of the first factor did. Five of the items belonging to the relational aggression factor were found to be consistent with oppositional defiant behavior problems, and two of them were consistent with attention deficit hyperactivity problems. The other aggression items did not meet the authors' criteria for consistency with DSM categories. Thus, the direct aggression factor resembles a fairly specific DSM-IV diagnosis of conduct disorder, whereas the relational factor resembles oppositional defiant disorder.

In order to settle this issue within our proposed theoretical model, we engaged three experts in studies of aggression to re-classify the existing items of the Achenbach system contained in aggression and delinquent behavior [60]. These re-classifications were done independently and blindly. There was 90% concordance between the three raters. Three items could not be classified. The resultant "hot and "cold" aggression subscales had a Cronbachs's alpha of .75 and .82 respectively. The new scales and the existing Achenbach "aggression" and "delinquency" scales correlated highly significantly and above 0.9. Thus, a decision was made to use the original YSR scales to preserve norms and continuity, and having established that for our purposes YSR delinquency would be now a proxy for "cold aggression - PIP", while YSR aggression would be a proxy for "hot aggression- RADI".

## The Empirical Testing of This Approach in Samples of High Ecological Validity

In this section, we will summarize work by our group of clinician-researchers that seeks to establish convergent, discriminate and predictive validity of the proposed bipartite model [60,61].

The studies are available in a recent publication that also provides fuller access to measures, analyses, and results [62]. In order to establish a basic rates of prevalence of PIP and RADI in a normal high school population, Steiner and colleagues [61] examined the characteristics of subjects standardized scores in the top two percent of the distribution in the YSR Version 1991 [56] aggression and delinquent behaviors dimensions respectively, as well as the overlap between the two dimensions. These analyses were performed in a previously described high school sample (N = 1434, 44% boys, ethnically diverse; mean age 16, SD = 1) [63]. This is a sample of students from two suburban high schools who completed self-report measures of demographics and the YSR. The demographic Facts About You scale [63], also reports on subjects self reported happiness with themselves, their defensiveness on a Likert scale ranging from 1-9, with nine being the happiest or most defensive. Age normed means are available.

Using these tools in this sample there were several interesting findings. 12% percent of these youth presented with problems in RADI aggression only; 9% with problems in PIP aggression only; and 5% with combined problems. As expected, boys were more likely to have problems with all forms of aggression combined, than girls (27% vs. 20%). In addition, the distribution of PIP vs. RADI and their combination was also different between genders. In all categories, boys surpassed girls (RADI only 12% vs. 10%, PIP only 10% vs. 6%, and combined 5% vs 4%; (Χ^2 ^= 12.3; df = 3, p = 0.007). But, as one would expect from a population based sample, most high school students (73-80%), regardless of gender, did not have problems with any form of aggression.

In order to examine the connections between the two forms of aggression, age, happiness, defensiveness and psychopathology, we performed Pearson's Correlation coefficients between the relevant variables. The two forms of aggression, RADI and PIP, correlated significantly with each other: Person's r=.44, p < .001, showing that to some extent these two forms of aggression are related, although the degree of overlap only accounted for about 16% of the variance. Thus, it seems that most high school students do not have problems with these two forms of aggressive behavior, however; there is a small number that have problems with both forms. Importantly, there are subsets of youths that have problems with one form or another, supporting the argument that these two subtypes can be differentiated on a descriptive and behavioral level. In the same study, the authors also tested differential associations of these forms of aggression, and found that, by and large emotional charged RADI aggression had consistently stronger correlations with the other YSR subscales of psychopathology (Pearson r's ranging from 0.38 to 0.62, with a mean of 0.50, all p's < 0.001). The strongest correlation was with Attention Problems (0.69), but Anxiety and Depression also showed a highly significant association of 0.46. By contrast, emotionally cold aggression showed more moderate associations (range 0.28 to 0.42, with a mean of 0.33; all p's < 0.001). The strongest association was with thought problems (r = 0.42).

In order to examine unique contributions of these variables onto each subtype of aggression, all YSR variables were entered into a linear regression model, along with control variables, such as age, gender, defensiveness and general happiness. The two subtypes of aggression were both significantly predicted, but by a different profile of independent variables (RADI r squared of .55 (F = 140; p < .001). In addition to PIP, all the other psychopathology scales made unique contributions as well. The most significant facilitating contributors were, in descending order: symptoms of anxiety and depression, attention and thought problems. Somatic complaints and social problems contributed more modestly. Withdrawal, Age and Happiness were protective, (i.e. stood in a negative relationship to the presence of RADI aggression). The independent variable set for PIP also resulted in a significant formula (r squared .40; F = 77.03, p < .001), and again, RADI, the other form of aggression also contributed most to the presence of PIP. However, the remainder of the independent predictors were different in the case of PIP. Attention problems and withdrawal, both were positive predictors of PIP. Most importantly, anxiety and depression was a protective factor against PIP aggression, as were being defensive and happy. Youths with problems in PIP aggression were not anxious and depressed (i.e. emotionally compromised). They had trouble with attention and tended to withdraw. All these analyses in this large and diverse high school sample support the contention that while there is overlap between RADI and PIP constructs, several important differences between them emerge. Particularly noteworthy is the change in relationship between anxiety and depression: a facilitator for RADI, they become protective against PIP. Youth who struggle with emotional upheaval are more likely to become emotionally aggressive as the model described above would posit.

These results immediately raise the question as to whether similar frequencies and relationships can be found in incarcerated youths, who have well documented problems with aggression in all forms and, as mentioned above, with psychopathologies, especially trauma related psychopathology. Finding similar separations between these two subforms of aggression in such samples would considerably strengthen the argument that this typology is ecologically valid. One also would hope that these manifestly disordered youths would show much higher levels of disturbance on the parameters measured in the high school study, showing that the model also has discriminate validity. The following studies used the magnifying lens of manifestly and chronically very aggressive youths, (i.e. a sample of incarcerated boys and girls) [9,61].

Using a previously described data base of 790 consecutively admitted youths [9] incarcerated in the California Youth Authority, we oversampled females (N = 140, 18%) in order to be to be able to examine gender effects. The mean age was 18 ± 1.2 years, (range 13-22). The ethnic distribution of the sample included Whites (N = 130, 17%), African-Americans (N = 224, 28%), Hispanics (N = 374, 47%) and Other (N = 60, 8%). This was a highly morbid sample by structured interview (SCID); excluding conduct disorder or oppositional defiant disorder, 88% (N = 571) of male and 92% (N = 129) of females had a psychiatric disorder in the prior year. Greater than 80% of both males and females met criteria for a substance use disorder. For this study, there was an expanded sets of measures available, which have been described in great detail elsewhere (9). The Achenbach YSR, 1991 version, [56]; The MAYSI [64]; the WAI - Weinberger Adjustment Inventory [65], and the Drug Experience Questionnaire [66]. Our choice of measures was driven by the findings in the high school sample and other previous work with incarcerated youths where we were able to show that these measures all had age appropriate norms. We and others were able to show that they have concurrent [65] discriminate [67] and predictive validity [68,69] in this severely compromised population.

**Figure 1:**
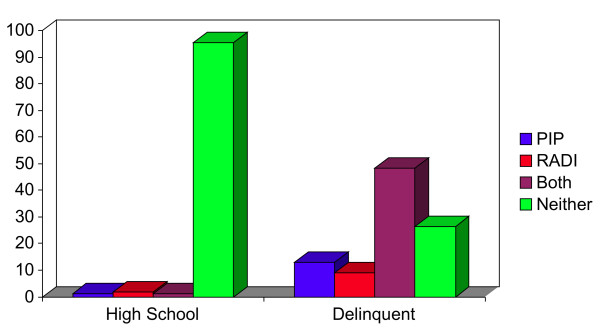
**Percent of clinically significant RADI and PIP aggression in High School Students and Delinquent Youth on Relabeled YSR Scales (Chi Square = 1975; DF 3; p < 0.0001)**.

The use of the MAYSI permitted us to examine more specifically the effects of traumatic incidents and Drug and Alcohol Abuse on our YSR variable of aggression subtypes. In addition, the WAI in turn provided us with trait measures of happiness to retain the parallel results to the normal sample. The results of this study are juxtaposed to our high school result in Figure 1.

What is immediately apparent in comparing the normal and delinquent adolescents is that the normal high school sample is very distinct on these aggression dimensions from the incarcerated sample, all in the expected beneficial direction. Most incarcerated youths have problems on both dimensions (48%), and only 28% have problems with neither. RADI aggression problems as measured by the YSR are more prevalent in delinquents than normal adolescents, (11% vs. 14%) and in the PIP dimension, 4.2% normals report problems, as opposed to 21% in the delinquents (Χ^2^= 487.4; df = 3; p = 0.0001). As was to be expected, the results confirm our hypothesis that these problems would be significantly more common in delinquents.

We also reported gender effects on both subtypes of aggression in incarcerated girls and boys. Overall, fewer boys are problem free than girls (77% vs. 73%). Boys have more problems with PIP aggression (14% vs. 5%) while girls report higher levels of RADI aggression (19% vs. 9%). This is of special interest, as we have reported that girls have almost twice the rates of psychopathology in this population, especially trauma related psychopathology [68]. Thus, we would expect delinquent girls to have more problems with RADI.

Reporting on the associations between psychopathology and the subtypes of aggression, a distinct picture emerges: Both forms of aggression correlate significantly with each other, more strongly in the delinquent sample than in the normal high school adolescents (Pearson's r = 0.53, p < .001). But even with this stronger association, each subtype of aggression accounts only for about 26% of the variance in the other. This supports the proposed separation of the RADI and PIP subtypes, even in this extremely compromised sample.

Both forms of aggression also correlate significantly with all symptoms subscales of the YSR. In the aggregate, as in the normal sample, RADI aggression continues to show stronger correlation coefficients than PIP aggression with measures of psychopathology. (RADI Pearson's r mean 0.42, range from 0.27 to 0.53; versus PIP Pearson's R mean 0.26, range from 0.18 to 0.39). Our addition of the MAYSI also permitted us to expand the correlations to Traumatic Experiences and Alcohol/Drug Use - both additional subscales which we did not have available in the normal high school sample. As we expected, RADI showed a stronger relationship than PIP with traumatic experiences (r = 0.36 vs. 0.28, both p's < 0.001). Interestingly, the pattern was reversed for the subscale Alcohol/Drug Use: PIP aggression has the stronger relationship with this subscale than RADI (r = 0.41 versus 0.27, both p's < 0.001). In incarcerated youths, higher levels of PIP aggression is positively associated with more abuse of alcohol and drugs. This relationship has not been reported before and should be explored further. Entering all these variables, along with control variables into a linear regression to find unique contributions of these variables onto each subtype of aggression, we entered them into a linear regression, expanding the predictor variables by the MAYSI subscales of traumatic events and drug and alcohol abuse.

This procedure produced distinct predictor formulas for the two subtypes of aggression just as they had in the normal high school sample. In addition, the independent predictor formulas between the normal high school sample and the delinquent sample also remained very similar. The YSR psychopathology subscales, augmented by the two MAYSI subscales of trauma and alcohol/drug abuse, and the control variables of happiness, age, gender and defensiveness resulted in an r squared of 0.55 (F = 140; p < 0.001) for RADI aggression. The most significant facilitating unique contributors were, in descending order: PIP aggression, symptoms of anxiety and depression, and thought problems. Withdrawal and MAYSI Alcohol and Drug abuse had a protective effect, meaning that youth experiencing problems in these domains were less likely to manifest problems with RADI aggression. By contrast, PIP predictors also resulted in a significant formula (r squared 0.40; F = 77.03, p < 0.001). The most important unique facilitating contributions came from RADI aggression, Attention problems, and Thought problems, and the MAYSI alcohol/drug abuse variable, and anxiety/depression, in contrast to RADI aggression, and just like in the normal high school sample, had a protective effect. This means that incarcerated youths who were anxious and depressed were less likely to report problems with PIP aggression. Traumatic experiences did not make any unique contribution to either form of aggression in incarcerated youths. We take this to mean that we are dealing with a ceiling effect, given that almost 80% of these youths reported non-normative untoward events. Most of the traumatic contribution is probably contained in the reports of anxiety and depression, which were shown to be in the opposite relationship for PIP and RADI, just like in the high school sample. Symptoms of anxiety and depression facilitate RADI problems, as the model presented above would posit, while they lessen the chances that an individual reports problems with PIP aggression. The consistency of findings in these two relatively large adolescent samples with such different backgrounds is encouraging.

## Implications for Treatment

The subtyping of aggression presents a new opportunity to reconsider our approach to treatment for disruptive behaviors in children and adolescents. A complete review of the treatment literature is beyond the scope of this review. We will only focus briefly on the implications of our findings thus far for the use of medications, psychotherapy and sociotherapy. It may well be that the two subtypes of aggression will have differential treatments. The PIP type will probably need interventions which help the child learn alternative ways of achieving desired outcomes, and a means to learn social norms other than aggression in a more "top down" oriented approach. There have been some encouraging effects of the application of Dialectic Behavioral Therapy, Cognitive Behavior Therapy and Parent effectiveness Training in youths with significant psychopathology [70].

We would expect these techniques to work for both forms of aggression, in contradistinction to psychopharmacology, which targets symptoms on more dedicated neurocircuitry and systems. Perhaps the RADI type which is increasingly being shown to run on more dedicated circuits in close connection with the threat detection system, will benefit more from a "bottom up" approach, as they seem prime candidates for medication treatment. In one consensus paper on treatments from the AACAP-Stanford-Howard Workgroup on Maladaptive Juvenile Aggression [27], we concluded that there are different treatment needs for children characterized as one or the other aggressive subtype. The workgroup felt that reactive children displayed more social skills deficits and were likely to face experienced psychosocial problems later in development. By contrast, while proactive children had better social skills, they tended to end up in situations where their aggression was reinforced, and in fact might even lead to desired goals. It was felt that this formulation led to a poorer prognosis for proactive children and youth, and also pointed to a more comprehensive, top down type intervention, such as is presented by the behavioral therapies cited above.

To address some of these issues in further detail and specifically to contend with the rising use of psychotropic medications for the treatment of childhood aggression, the Food & Drug Administration (FDA) convened an expert panel to develop guidelines for the use of medications in the context of impulsive aggression [37]. The panel found impulsive aggression to be factor across a range of psychiatric disorders and that its construct seems to be similar across these disorders. They further concluded that the current research should focus on well designed studies that look at the presentation of impulsive aggression within existing DSM-IV classified disorders, and that clinical trials data from these studies can inform the use medications. The panel use examples of DSM diagnoses of ADHD, autism, PTSD and bipolar disorder within which impulsive aggression could be effectively studied. The panel gave explicit guidelines as to how to design these studies, and these should form the basis for future research.

As a final test of the RADI/PIP division of aggression and its disturbances, we explored what its effects are by re-analyzing an existing data base along the lines suggested by the discussion so far in our recent publication [71].

Fifty-eight delinquent males, were treated with low or therapeutic doses of Divalproex Sodium (DVP), in a randomized clinical trial, double blind and placebo controlled, which we have published previously [72-74]. Subjects were subtyped into High Distress Conduct Disorder (HDCD) and Low Distress Conduct Disorder (LDCH) which corresponds with individuals who had committed highly emotionally charged (RADI) and carefully planned, unemotional (PIP) aggressive acts respectively. Results showed that response rates to DVP were significantly higher among HDCD subjects (64%) than among LDCD subjects (22%) in the high-dose treatment group (p = 0.03). These results support the utility of antikindling agents such as DVP in treating patients with disorders characterized by the RADI pattern of aggression, including those with severe CD. They also lend further support to the distinction between these two forms of aggression by showing that they predict different distinct patterns of response to medications that reduce negative emotionality [71].

Analyses of this kind can probably applied to other important data bases which report on the psychopharmacology of aggression. As in this previous study, we would expect that other agents, such as atypicals, SSRI's and SNRI's and mood stabilizers should show efficacy predominantly against RADI aggression, in the context of other psychopathologies, such as bipolar disorder, depression, anxiety disorder and Posttraumatic Stress Disorder [40,75].

Our redefined subdivision of aggression most likely also has important implications for the taxonomy of disorders of aggression. The current labels of Oppositional defiant disorder and conduct disorder, while having some congruent and discriminant validity, suffer from the main problem that they are too encompassing and vague, with little positive predictive value. Furthermore, they rarely lead the clinician to any specific interventions along the lines suggested by the current subdivision above. Reshaping the descriptive diagnostic criteria to create two diagnostic spectrums, along the lines of acute, chronic and low grade disorders of RADI and PIP aggression, respectively, might make these labels considerably more useful, as we have argued in a previous publication [16]. The developing differential neurocognitive profiles of the two spectra also supports this argument [21].

## Conclusion

Our findings support the existence of two relatively distinct forms of aggression in large, modern samples of normal and delinquent youths of high ecological validity. The proposed subtyping of aggression into PIP and RADI has additional support from history, the law and cognitive neuroscience. We are able to show gender effects and modest age effects. We also are able to show that of the two forms of aggression, the emotionally hot RADI form has a much closer relationship to disturbances of emotional functioning, as in PTSD, Dissociative Disorder, Bipolar Disorder. These findings suggest that we should pursue further subtyping of disruptive behavior more systematically, as it appears that these acts of hot emotional RADI aggression are a legitimate target for psychopharmacological and other trauma specific interventions. Re-analyses of existing data sets shed new light on the positive contributions this further distinction of disruptive behavior can make. In the case of the diagnostic labels of disruptive behavior disorders, we are in need of finer distinctions that can lead clinicians to more specific clinical interventions which in turn, promise to improve hitherto unimpressive treatment outcomes of delinquents and patients with disruptive behavior.

## Competing interests

The authors declare that they have no competing interests.

## Authors' contributions

This review was designed and written by HS. MS and NK have significantly contributed in terms of the conception of the article and the acquisition of data. JH and BP were essentially involved in drafting the manuscript and revised it critically. CC, JB and RH prepared the analysis and interpretation of data and contributed important intellectual content to the manuscript. All authors read and approved the final manuscript.
